# Applications of Nanoparticle-Antibody Conjugates in Immunoassays and Tumor Imaging

**DOI:** 10.1208/s12248-021-00561-5

**Published:** 2021-03-14

**Authors:** Xinhao Lin, André O’Reilly Beringhs, Xiuling Lu

**Affiliations:** Department of Pharmaceutical Sciences, University of Connecticut, Storrs, Connecticut USA

**Keywords:** antibody conjugates, immunoassay, nanoparticle, tumor imaging

## Abstract

Modern diagnostic technologies rely on both *in vitro* and *in vivo* modalities to provide a complete understanding of the clinical state of a patient. Nanoparticle-antibody conjugates have emerged as promising systems to confer increased sensitivity and accuracy for *in vitro* diagnostics (e.g., immunoassays). Meanwhile, *in vivo* applications have benefited from the targeting ability of nanoparticle-antibody conjugates, as well as payload flexibility and tailored biodistribution. This review provides an encompassing overview of nanoparticle-antibody conjugates, from chemistry to applications in medical immunoassays and tumor imaging, highlighting the underlying principles and unique features of relevant preclinical applications employing commonly used imaging modalities (e.g., optical/photoacoustics, positron-emission tomography, magnetic resonance imaging, X-ray computed tomography).

## INTRODUCTION

Over the past decades, nanoparticle systems have attracted significant attention in biomedical research and applications. Nanoparticles are within the nanometer-size range with the potential to tailor biodistribution *in vivo*, generally designed for improved drug delivery and biocompatibility. Notably, various nanoparticle systems have been studied as a means of targeting and increasing the accumulation of their cargo in tumor tissues, taking advantage of the tumors’ leaky vasculatures, which allow nanoparticles to extravasate out of the vasculature and be retained in tumor tissues. This tumor physiology-based phenomenon is known as the enhanced permeation and retention (EPR) effect. Nanomedicines may offer increased safety profiles, as healthy tissues are less exposed to the particle’s payload due to their limited biodistribution *in vivo*, but not necessarily greater dose accumulation at the tissue of interest. It has been recognized that the nanoparticle delivery efficiency to tumors is minimal, which can significantly impair product performance as the absolute amounts of nanoparticles extravasating into tumors may not be enough to achieve their purpose. Recent surveys of preclinical data from xenograft tumor models have demonstrated that less than 2.25% (mean) of the injected nanoparticle-based dose accumulates in solid tumor tissues (1,2). Furthermore, the EPR effect is known to be inconsistent and variable inter- and intra-individual (3), which is one of the reasons phase II/III clinical trials with nanomedicines for cancer therapy have shown higher-than-expected failure rates (3–5). In this sense, researchers have tried to address these passive targeting issues by employing active targeting strategies, especially using antibodies or antibody fragments. Cellular internalization can be significantly improved; however, antibody-nanoparticle conjugates would need to overcome the same delivery challenges that non-targeted nanoparticles face accumulating in tumor tissue, travel through tumor stroma, then the targeting modality could enhance the interaction of nanoparticles with tumor and improve the tumor specificity. For drug delivery, the antibody-nanoparticle conjugates exhibited limited improvement in the delivered amount of drug to the targeted site as well as treatment outcome, despite many attempts in the past years.

Recently, nanoparticle-antibody conjugates have been employed in the development of diagnostic and imaging platforms for both *in vitro* and *in vivo* applications. Combining nanoparticles with antibodies enables improved *in vitro* diagnostics, namely immunoassays, by leveraging electron charge oscillations in particles for optical enhancement and enhancing sensitivity. For *in vivo* applications, the vast majority of the research conducted with nanoparticles for diagnostic purposes focuses on tumor imaging. Several *in vivo* imaging modalities can observe the dynamic changes of the tumor accumulation of nanoparticles and allow for non-invasive detection of overexpressed tumor surface antigens, discrimination of tumor malignancy, and determination of suitable therapeutic strategies. Besides, they can aid in identifying the intratumoral distributions of specific markers through the use of ultrasmall nanoparticles conjugated with an antibody fragment. This review will highlight the recent progress on the applications of nanoparticle-antibody conjugates in immunoassays and tumor imaging.

## PREPARATIONS OF ANTIBODY NANOPARTICLE CONJUGATE

### Antibody as a Targeting Agent

**Fig. 1:**
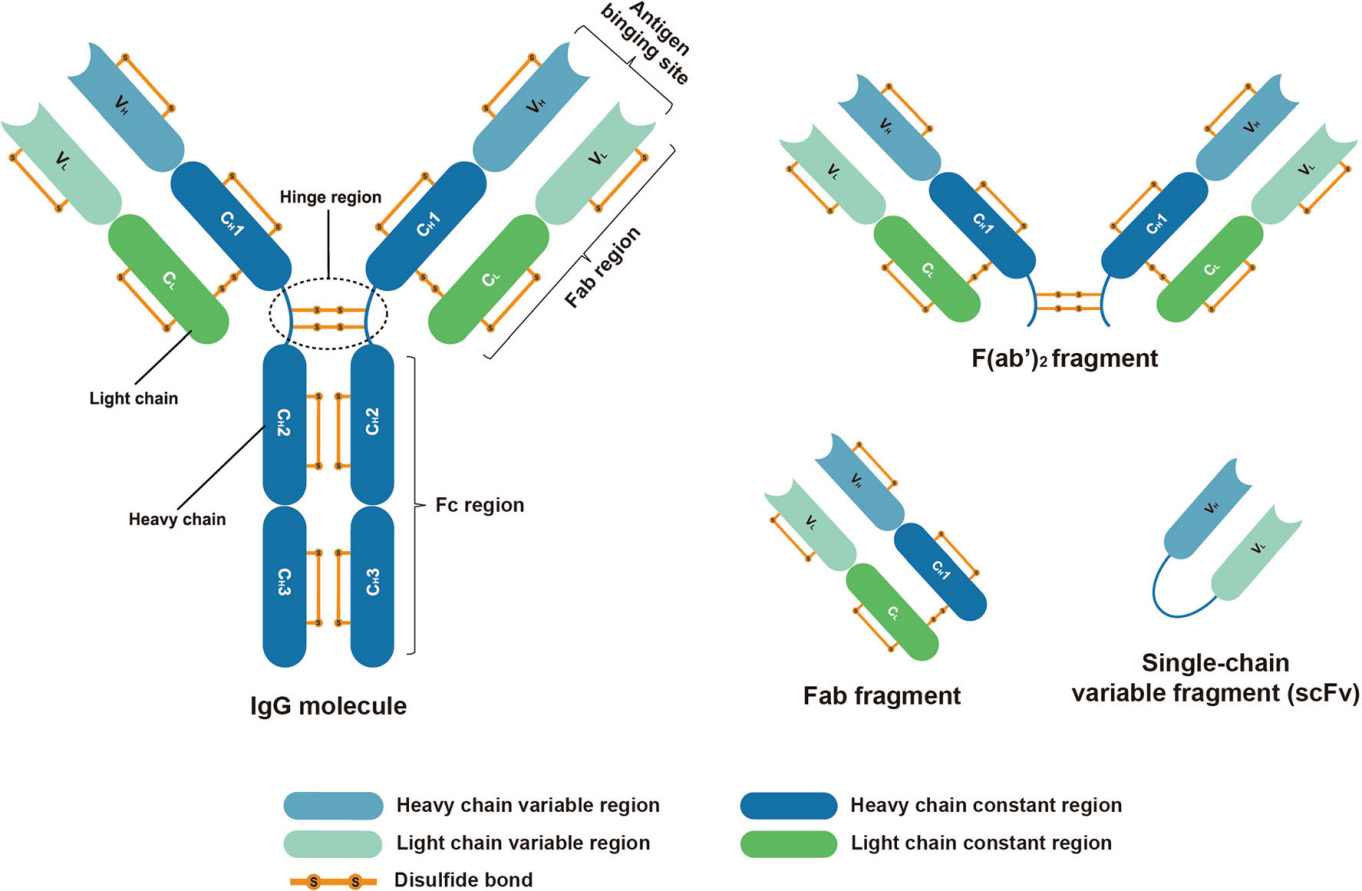
Structure of IgG molecule and its fragments: F(ab’)_2_ fragment, Fab fragment, and single-chain variable fragment (scFv)

Antibodies can be found on the surface of B cells and can act as B cell antigen receptors (BCR) or be secreted to bind and neutralize their target antigens (6). Currently, antibodies are widely used in clinical practice, especially in cancer therapy. They can not only be used in direct antibody treatment but also serve as a targeting ligand (7). Antibodies are composed of a 50 kDa heavy chain and a 25 kDa light chain (Fig. 1). Based on the structures and properties of the C regions, antibodies can be classified as immunoglobulin M (IgM), immunoglobulin D (IgD), immunoglobulin G (IgG), immunoglobulin A (IgA), and immunoglobulin E (IgE) (6). Among the five isotypes, the IgG antibody is the most abundant in human plasma. It can be further classified into four subclasses, IgG1, IgG2, IgG3 and IgG4, according to the differences in amino acid composition in the Fc region (8).

In IgG antibodies, each of four polypeptide chains has a variable (V) region responsible for binding antigen and a constant (C) region. They can be divided into two fragments based on their functions, which are the antigen-binding fragment (Fab) and the fragment crystallizable (Fc) region (9). Different enzymes may be employed to divide IgG antibodies into smaller fragment.

Employing recombinant antibody technologies, single-chain variable fragment (scFv) can be achieved, which includes the variable heavy and variable light domains. With two antigen-binding domains linked by a flexible peptide, scFv can provide excellent affinity and alteration of specificity to specific targets (10), while displaying smaller molecular size. The structure and fragments of IgG antibody are shown in Fig. 1.

### Conjugation Methods

Antibody functionalization on nanoparticle surface includes mainly two conjugation methods: adsorption and covalent binding (11,12). The conjugation efficiency refers to the amount ratio of conjugated antibodies to the total amount of antibodies, which indicates the ability of antibodies to be immobilized (13). Depending on the conjugation method employed, immobilization of antibodies on the surface of nanoparticles can be site-specific or non-site specific. Moreover, binding sites determine the orientation of the antibodies (14). The ideal orientation of antibodies in nanoparticle-antibody conjugates occurs when the Fc region is attached to the surface of the nanoparticles, enabling the antigen binding sites within the Fab regions to interact with the antigen appropriately (15). Opposite to oriented conjugation of the antibody, random antibody orientation can be achieved in numerous common approaches (16). Still, a reduction in binding ability must be considered if random orientation is the case.

### Adsorption

Adsorption, including physical and ionic, is one of the simplest conjugation methods. The antibodies attach to the material surface owing to intrinsic surface interactions, such as Van der Waal forces, electrostatic forces, hydrophobic interactions, and hydrogen bonds. Using this simple and gentle method, fragile antibodies can be immobilized onto surfaces with limited damage (17). On the other hand, this approach often results in diminished physical stability compared with covalent binding and, therefore, conjugation efficiency and retention may be reduced (18). Furthermore, due to the intrinsic lack of specificity associated with physical and ionic interactions, random antibody orientations are favored.

### Covalent Binding

Accompanied with high stability and excellent reproducibility, covalent binding is preferred because covalent bonds are less susceptible to disassembly (19), leading to stronger conjugation with orderly antibody orientation when compared with adsorption. Carbodiimide chemistry and maleimide chemistry are the most commonly used covalent binding approaches for nanoparticle-antibody conjugation.

#### Carbodiimide Chemistry

A crosslinking method using carbodiimide compound, such as 1-ethyl-3-(3-dimethylaminopropyl) carbodiimide (EDC) and *N*,*N*′-dicyclohexylcarbodiimide (DCC), to crosslink carboxylic acids to primary amines (20,21). The amine groups of antibodies can be abundant, and they can be very reactive without further chemical modification (22). During the reaction, *N*-hydroxysuccinimide (NHS) or *N*-hydro-xysulfosuccinimide (sulfo-NHS) are often used because they can increase the EDC-mediated coupling efficiency. The existence of NHS or sulfo-NHS produces a more stable intermediate that helps to prevent intra and intermolecular crosslinking of the antibody (23–25). Based on its mechanism, carbodiimide chemistry can lead to a random immobilization of the antibodies on the nanoparticle surface and affect antibodies' biological activity and targeting ability because most of the amine groups in the Fab region can be reactive (22,26).

#### Maleimide Chemistry

A site-selective conjugation approach that involves binding through the sulfhydryl groups (-SH), also called thiol group, of the antibody. Sulfhydryls exist in proteins on the side chain of cysteine amino acids. Pairs of cysteine sulfhydryl groups are coupled by disulfide (-S–S-) bonds *via* an oxidative process (27). For IgG antibodies, disulfide bonds are usually present on the hinge region of the antibody structure. The reduction of disulfide bonds can cleave the antibody into monovalent halves without changing the 3D structure antigen-binding efficiency (28,29). However, only free sulfhydryl groups can be used in maleimide chemistry, requiring the reduction of disulfide bonds by sulfhydryl-addition reagents or reducing agents. Sulfhydryl-addition reagents, including Traut’s reagent (2-iminothiolane) and *N*-succinimidyl *S*-acetylthioacetate (SATA), can modify the amine group of lysine residues with thiol groups (30). However, by increasing the number of thiol groups, site-selectivity may be lost, negatively impacting antibody interactions with its target. Like DTT and BME, reducing agents can cleave native disulfide bonds without adding new thiol groups and are preferred from a conformational perspective. SMCC, Sulfo-SMCC, and their PEGylated analogs are the most popular crosslinking reagents in maleimide chemistry.

#### Click Chemistry

This refers to a group of simple chemical reactions with stereospecificity and high efficiency (31,32). Its selective, orthogonal properties to most known reactions and generating minimal byproducts make it an excellent platform in biomedical applications (33). Cycloadditions, nucleophilic ring-openings, carbonyl chemistry of the non-aldol type, and additions to carbon-carbon multiple bonds are the four classifications of click chemistry (34). Cycloaddition reaction, as the most widely used click chemistry methods in nanoparticle-antibody conjugates, includes copper-catalyzed azide–alkyne cycloaddition (CuAAC) reaction, strain-promoted alkyne-azide cycloadditions, and inverse electron demand Diels–Alder reactions (18).

## ENHANCING IMMUNOASSAY SENSITIVITY USING NANOPARTICLE-ANTIBODY CONJUGATES

Sensitive and quick methods for immunoassays are demanded in a wide range of fields, including diagnostics, therapeutics, and food safety. In essence, these are widely used *in vitro* assays conducted with biological samples to investigate analytes of biochemical interest. Due to its reliance on antibody binding mechanisms, immunoassays have been acclaimed for their relative sensitivity and specificity, but improvements are still warranted. Detection and quantification of small amounts of biomolecules can be optimized by the application of nanoparticles with adjustable surface chemistries, tunable optical properties and biocompatibility (35). It stands to reason that nanoparticle-antibody conjugates are great candidates to improve the sensitivity in immunoassays. Based on mechanisms and methods, immunoassays leveraging nanoparticle-antibody conjugates can be performed using a variety of methodologies, including surface plasmon resonance (SPR), localized surface plasmon resonance (LSPR), surface-enhanced Raman scattering (SERS), and electrochemistry and fluorescence (36). SPR and LSPR are especially relevant for nanoparticle-antibody conjugates employed in immunoassays due to their potential in signal amplification.

### Surface Plasmon Resonance

Metal nanoparticles, especially gold (Au) nanoparticles, with their outstanding plasmon resonance properties, can be employed as signal enhancement tools for SPR (37). Plasmons are defined as collective oscillations of free electrons present in metals at a well-defined frequency (38). This is attributed to the localized electromagnetic fields that occur at nanoparticle surfaces. Au nanoparticles have been widely reported for SPR signal enhancement due to their abundance of easily polarizable conduction electrons, a requirement for preferential interactions with electromagnetic fields (39). Due to coherent conduction electron oscillation, an electron is displaced from the nuclei originating a surface charge distribution which is subsequently restored due to Coulomb attraction forces (40), and these individual collective oscillations are characterized as SPR. Most importantly, plasmon excitation is a surface phenomenon (39), therefore particularly fostered on metal nanoparticles due to their high surface area when compared with bulk metal materials (40). In general, a clear understanding of SPR in metal nanoparticles is still lacking, but significant efforts in elucidating the impact of particle size and geometry in SPR have been conducted (40). Furthermore, SPR oscillations are highly sensitive to any changes of surface boundaries, such as adsorption of molecules onto the surface, which is a characteristic that is leveraged when employing SPR for analytical purposes in antibody-nanoparticle conjugates (41). With immobilization of antibodies on the surface of Au nanoparticles, sandwich immunoassays can be achieved for large molecules with multiple binding sites (42), while taking advantage of the SPR effect. Sandwich immunoassays are typically defined as a specific antibody assay where matched antibody pairs are used, one for analyte immobilization and another for detection.

**Fig. 2.**
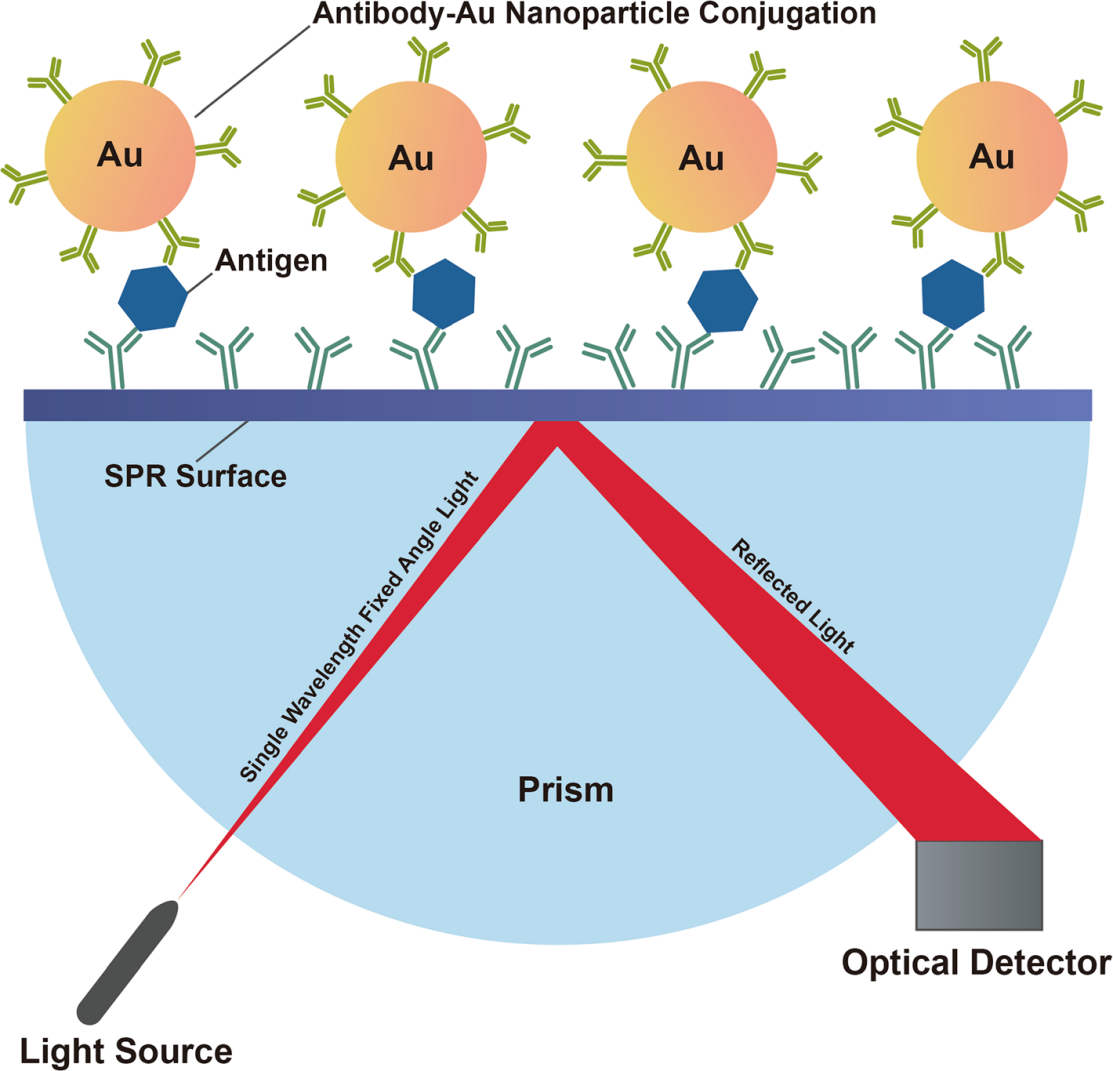
Schematic representation of a sandwich immunoassay leveraging nanoparticle-antibody conjugates for surface plasmon resonance (SPR)

Au substrate and antibodies can be used to fabricate a biosurface, which functions as the bottom of the sandwich providing a stable layer for amplification of the SPR signal. A typical binding structure of a sandwich immunoassay leveraging SPR is shown in Fig. 2. Analytes are captured by nanoparticle-antibody conjugates with an Au substrate in the middle. Gold-thiol interactions using 2-mercaptoethylamine (MEA) (43) and a self-assembly method using succinimidyl-terminated propenthiol (DSP) (44) are two methods used in biosurface fabrication. Nanoparticle-antibody conjugates, as the top of the sandwich, can be produced by different linking strategies including gold–thiol interactions (43), 3,3′-Dithobis (sulfosuccinimidyl propionate) (DTSSP) as a bifunctional crosslinker (45) and PEGylation (46).

In conclusion, the signal amplification produced by the nanoparticle-antibody conjugates enables the reduction of the limit of the detection (LOD), thereby leading to a significant increase in the sensitivity.

### Localized Surface Plasmon Resonance

LSPR is generated by the interaction of light and noble metals capable of producing a collective oscillation of conduction band electrons when the size of the surface is much smaller than the photon wavelength. This phenomenon fosters non-propagating excitations, denominated localized surface plasmons, where the plasmon oscillation distributes throughout the entire particle volume, creating a mass-spring harmonic oscillator effect (41). Compared to traditional SPR, LSPR provides a more tunable wavelength, lower sensing volumes, and a more affordable cost for analytical purposes (47).

**Fig. 3:**
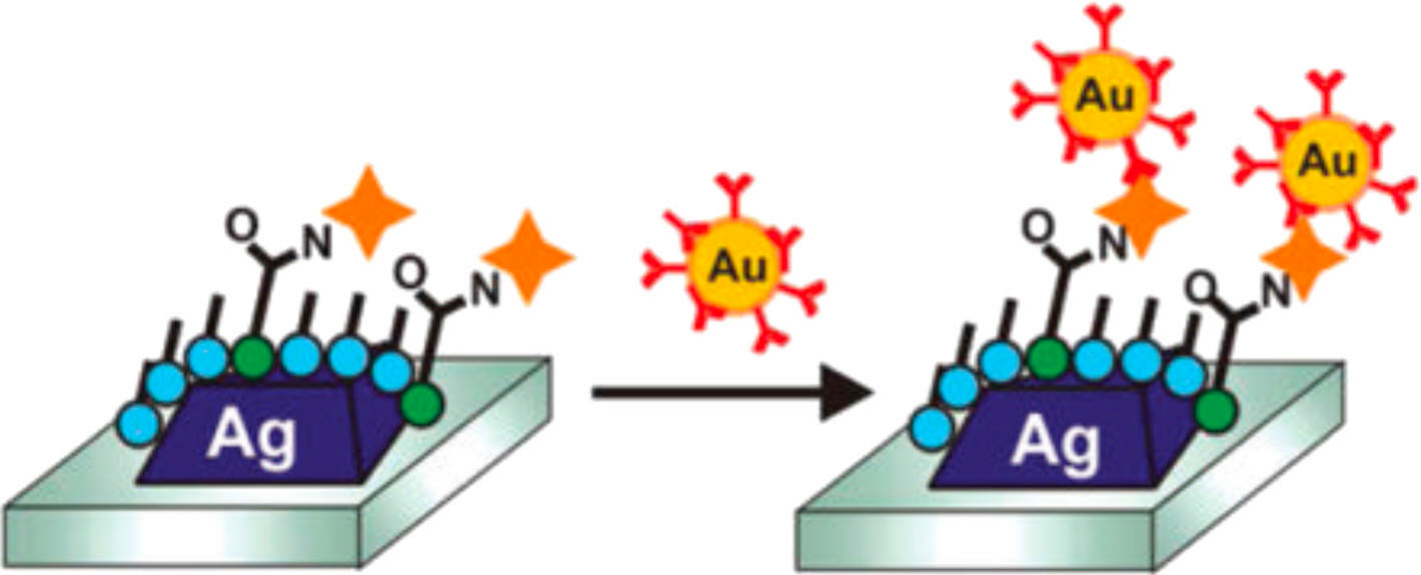
Schematic representation of an immunoassay leveraging nanoparticle-antibody conjugates for localized surface plasmon resonance (LSPR) (48)

Antibodies, acting as bioreceptors, can be carried by metal nanoparticles to improve the limits of detection for various analytes and increase the LSPR shift because of the changes in the local refractive index around the metal nanoparticle (48). A scheme of how nanoparticle-antibody conjugates work on LSPR is shown in Fig. 3. Instead of using Au substrate as a stable layer to amplify the signal, nanoparticle-antibody conjugates can recognize and bind to analytes, producing strong resonance absorbance peaks in the visible light range (380–700 nm), resulting in an LSPR peak shift.

Silver nanoparticles can be conjugated with antibodies to provide a peak shifting in LSPR. As Valdez reported, polyclonal antibodies for respiratory syncytial virus, which is a paramyxovirus that leads to mild, cold-like symptoms, can be functionalized on silver nanoparticles using 1-ethyl-3-(3-dimethylaminopropyl) carbodiimide (EDC) chemistry. The functionalized silver nanoparticles can interact with the respiratory syncytial virus and have a specific peak shifting at 60 min (50).

Overall, both silver and gold are excellent materials for nanoparticle manufacturing and have demonstrated suitable optical properties for analytical purposes. In general, plasmon resonance is considered insensitive to metal composition as the bulk plasma frequencies of noble metals are similar (51). Among the two materials, gold has been more widely popular due to its unique optical properties, low toxicity, and ease of chemical modifications and moieties attachment (52), justifying the prevalence of gold nanoparticles in LSPR applications. Being sensitive to the local changes of the dielectric environment around nanoparticles (53) and large color changes resulting from interparticle plasmon coupling (54,55) are strategies that can be utilized in LSPR combined with the conjugation of antibodies to gold nanoparticles. Prostate-specific antigen (PSA), which is being used as an antigen to screen patients for prostate cancer, can be detected by gold nanoparticles coated with anti-PSA antibodies. Gold nanoparticle-antibody conjugates enhanced the LSPR signal, and as a result, expand the dynamic range and improve the sensitivity (48). In general, antibody-functionalized gold nanoparticles have been shown to improve sensitivity up to 2.5-fold compared to blank gold nanoparticles, providing analytical performances at the ng/mL level for selected biomolecules (48,56–58).

Considering the complexity involved in antibody-nanoparticle conjugates, the conjugation stability may play a significant role in the analytical performance of this methodology and therefore must be addressed. For stability purposes, the most crucial factor is pH. The conjugate’s electrostatic attraction can be affected by pH-dependent flocculation if the antibodies are conjugated to the metal nanoparticles non-covalently (59). To protect the configuration and bioactivity of antibodies, pH should be adjusted to approximately the antibodies’ isoelectric points. Besides pH, antibody concentration during synthesis also matters as it can affect the conjugates' binding properties and stability (58). Therefore, proper efforts should be placed on developing the antibody-nanoparticle conjugate itself to ensure reproducible and consistent results.

## NANOPARTICLES AS VEHICLES FOR TUMOR IMAGING

### General Considerations

For *in vivo* applications, the vast majority of the research conducted with nanoparticles for diagnostic purposes focuses on tumor imaging. A variety of colloids have been widely used in imaging preclinically and more recently with limited clinical applications. Many types of nanoparticles, including sulfur colloid, albumin colloidal nanoparticle, and iron oxide nanoparticle, received approval from the U.S. Food and Drug Administration (FDA) for their application in radionuclide imaging and MRI (60). As one of the most critical types of nanoparticles used in imaging, metal nanoparticles, including gold, silver, and iron oxide nanoparticles, have drawn significant attention because of their targeting and ability to yield image contrast as imaging probes (61). Upon systemic administration, nanoparticles tend to accumulate in solid tumor tissues selectively. These principles have been the cornerstone of nanoparticle delivery to tumors for therapeutic purposes, and it also underlines the principles behind using nanoparticle-based contrast agents for tumor imaging. This perceived selective accumulation occurs due to (4,62):

1. *Enhance permeation and retention* (*EPR*) *effect:* tumor vasculature, especially concentrated at the tumor-host interface and within the stroma, does not mature properly and faulty vasculature allows easier extravasation of macromolecular structures into the tumor stroma. Poor lymphatic drainage kinetically entraps nanoparticles in tumor tissue, increasing their residence time at the site of interest
2. *Low volume of distribution* as the vast majority of the nanoparticle-loaded dose is retained within the blood vasculature (when compared with a small-molecule equivalent)
3. *Prolonged half-life in circulation* increases the likelihood of nanoparticle extravasation due to the probability
4. *Combination of EPR effect with long circulation* can universally enhance tumor accumulation

In reality, the assumptions mentioned above have been established over many years of preclinical studies using well-controlled tumor-bearing mouse models. Nowadays, an overall understanding is that the EPR effect is highly variable and preclinical tumor models usually fail to translate these challenges (as observed in human patients). This is shedding light onto the challenges of nanoparticle-based delivery to tumors of both imaging agents and therapeutic molecules. In general, nanoparticle-antibody conjugates have been portraited as a viable approach to increase the targeting-ability of nanoparticles and their retention at the tissue of interest. However, unless a specific vascular-targeting ligand is used, nanoparticle extravasation into tumor tissues through the EPR effect is still required for antibody-ligand interactions to occur. Therefore, most “active-targeting” nanoparticle systems will still be affected by inter and intra-individual variations in the EPR effect and will perform mainly by increasing nanoparticle-cell interactions and retention in the tumor tissue.

*In vivo* tumor imaging can be performed using a variety of modalities, whereas the most commonly used in the clinic include positron emission tomography (PET), magnetic resonance imaging (MRI), and X-ray computed tomography (CT). Nanoparticle-antibody conjugates can be used as active-targeting nanoparticle-based contrast agents for multimodality imaging and they may provide significant insights regarding tumor extravasation and “leakiness”. The vasculature leakiness is due to the abnormal and rapid growth of tumor vasculatures and is highly variable depending on the type and growth of tumors. Considering the intra- and inter-individual heterogeneity observed for the EPR effect in the clinic, it is genuinely considered a significant bottleneck hindering product performance *in vivo* (63). The ability of reliable and efficiently determined EPR levels in a patient *via* tumor imaging may provide a tool for patient pre-selection based on their likely response to a nanoparticle treatment *versus* free drug, increasing likelihood of performance success (e.g., therapeutic efficacy, imaging contrast, etc.). In fact, such an approach has been explored at the clinical level. For example, Merrimack Pharmaceuticals (USA) has administered ^64^Cu-labeled HER2-targeted PEGylated liposomal doxorubicin to screen metastatic breast cancer patients as part of their MM-302 clinical trial (64), followed by PET/CT imaging. Merrimack has found a 35-fold (0.52–18.5 %ID/kg) variation in tumor accumulation for these patients measured *via* PET, as an indication of EPR effect variability, and it allowed the classification of patients as a function of nanoparticle deposition in cancerous lesions based on a cut-off value extrapolated from preclinical studies (64). This approach could be generally adopted for clinical trials to select general patient populations for their likelihood to respond to a nanoparticle-based treatment as an imaging-guided therapy approach. In this context, nanoparticle-antibody conjugates can facilitate imaging by increasing retention at the tumor site. Alternatively, nanoparticle-antibody conjugates may also provide information regarding the presence and location of specific antigens of interest by targeting those within the tumor and allowing for real-time imaging without the need for biopsy and posterior analysis.

### Non-Invasive Detection of Overexpressed Tumor Surface Antigens

Although the overexpression of specific antigens on tumor surfaces has been documented, due to the variations among tumors as well as the expression level heterogeneity, validation of the targetability of the antibody nanoparticle conjugates through imaging provides critical indications to guide treatment strategies. Targeted nanoparticles containing imaging agents can be retained in tumor regions once reaching the area, given their strong affinities to specific tumor surface antigens, and exhibit enhanced signal contrast which provides the analyst with precise information regarding localization and density of such antigens within the tissue. A variety of imaging modalities, including optical imaging, photoacoustic imaging, and magnetic resonance imaging, have been used for this purpose.

**Fig. 4:**
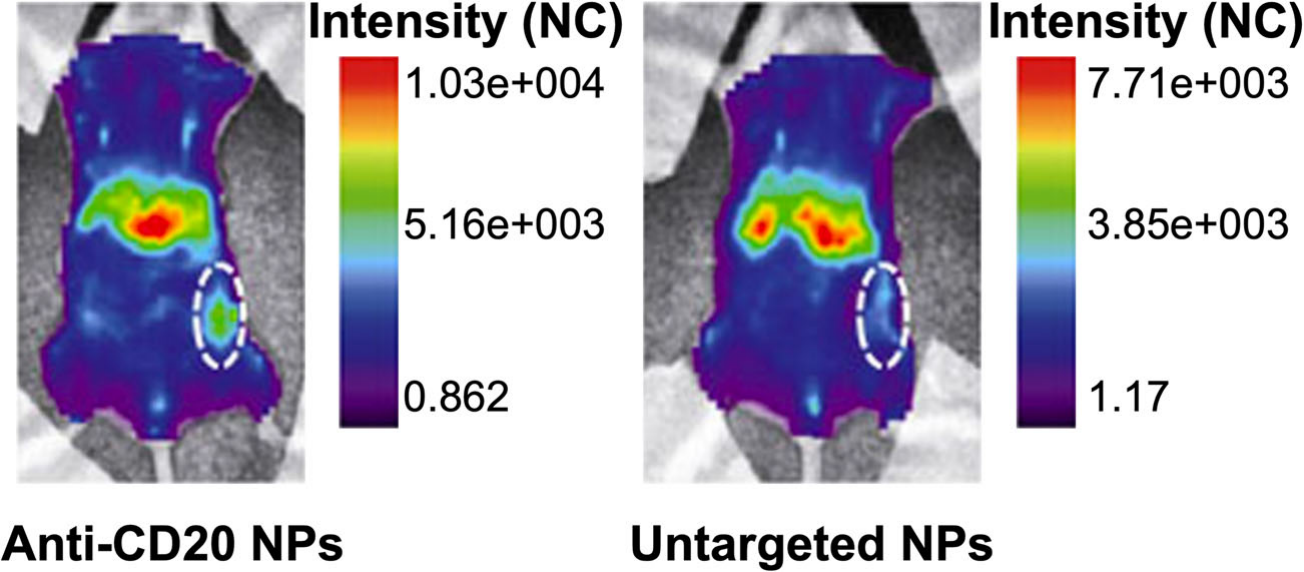
Whole-body fluorescence intensity distribution in a representative leukemic mouse 24-h post-injection of cy5.5-anti-cD20 NPs and cy5.5-untargeted NPs. The circles enclose the tumors (49)

Optical imaging is a non-invasive technique using luminescent or fluorescent reporter genes or injectable fluorescent or luminescent probes (65). Fluorochrome-labeled nanoparticles, with suitable retention properties, can accumulate in tumors, thus providing high-definition optical images. The use of antibodies further enhances its targeting feature. As shown in Fig. 4, Cy5.5-anti-CD20 nanoparticles (NPs) boost the improvement of signal-to-background ratios (7:1) comparing with Cy5.5-untargeted NPs. It provides more detailed morphology information of the tumor by a significant difference in fluorescence intensity from 24 h until 96 h after injection (49). A similar result was obtained from another research using Bevacizumab, a recombinant humanized monoclonal antibody directly against VEGF, as a target ligand to help iron oxide nanoparticles (IONPs) gain advantages in cancer imaging. Bevacizumab-IONPs conjugates yield a strong NIR signal at 48 and 96 h post-injection (67).

However, due to the limited penetration depth of optical signals, only a few studies have investigated the nanoparticle-antibody conjugates in optical imaging, and most of their applications are detections of overexpressed tumor surface antigens (68). Furthermore, optical imaging applications in the clinic are limited and may only provide information on superficial tumors.

Photoacoustic imaging is a hybrid biomedical imaging modality that delivers light energy and causes thermoelastic expansion of the tissues that absorb the light, and the tissue penetration is less limited compared with optical imaging (69,70), enabling applications beyond preclinical settings. Nanocarriers have been studied as imaging probes in photoacoustic imaging. There are two types of nanoparticles that have been investigated. One is metal nanoparticles, including gold, silver, and iron oxide nanoparticles (71–75). Due to their tunable optical properties and overall bright near-infrared emissions, these nanoparticles can be employed as imaging probe by themselves, without requiring the encapsulation of other optical imaging moieties (76). Gold nanostars conjugated with CD44v6 monoclonal antibodies (74), gold nanorods conjugated with anti-HER2 and -CXCR4 antibodies (71), and iron oxide nanoparticles conjugated with anti-HER2 antibodies (72) have been successfully used to image antigen overexpression due to their targeting-ability to specific tumor antigens. Another type of nanoparticle system widely used for this photoacoustic imaging preclinically is polymeric, mainly poly (lactide-co-glycolide) (PLGA) nanoparticles, which can carry a wide variety of near-infrared imaging agents (77,78). Metastatic lymph nodes were identified through CXCR_4_-SDF-1 interactions using photoacoustic imaging of indocyanine green and chemokine SDF-1-loaded PLGA nanoparticles (77).

MRI, a non-invasive clinical imaging technique, is widely used in biomedical imaging and clinical diagnosis. Paramagnetic or superparamagnetic contrast agents are administered prior to MRI as they can decrease the magnetic spin-lattice (T1) or spin-spin (T2) relaxation times of the protons on nearby water molecules and therefore increase sensitivity and resolution (79,80). Magnetic nanoparticles, especially superparamagnetic iron oxide nanoparticles, metallic nanoparticles, and bi-metallic nanoparticles, have unique magnetic properties and enable tracking through MRI (81). Superparamagnetic iron oxide particles, which are extensively studied as a diagnostic agent in different diseases, are T2-weighted MRI contrast agent and works by shortening T2 relaxation times in MRI. These nanoparticles could be conjugated with a variety of antibodies, achieving strong affinity with specific overexpressed tumor surface antigens, and enabling real-time imaging of the distribution and density of these antigens. Representative preclinical studies of nanoparticle-antibody conjugates for MRI purposes are shown in Table I, demonstrating the variety of overexpressed tumor surface antigens that have been studied as targets.

**Table I:** Relevant Examples of Nanoparticle-Antibody Conjugates and Their Applications in MRI

Name of nanoparticle	Disease/Application	Target of antibody	Type of contrast agent	Reference
Manganese oxide–mesoporous silica nanoparticles	Prostate cancer	Prostate-specific membrane antigen (PSA)	T1	(82)
Manganese oxide nanoparticles	Cancer (murine breast tumors)	Anti-CD105 antibody	T1	(80)
Gold nanocages modified with hyaluronic acid	Pancreatic cancer	Glypican-1	T1	(83)
Superparamagnetic iron oxide nanoparticles: Molday ION Rhodamine-B Carboxyl	PSMA positive prostate cancer cells	Prostate-specific membrane antigen	T2	(84)
Superparamagnetic iron oxide nanoparticles	Breast cancer	Her2-expressing MCF7/Her2-18 breast cancer cells	T2	(85)
Superparamagnetic iron oxide nanoparticles	Pancreatic cancer	Plectin-1	T2	(86)
Superparamagnetic iron oxide nanoparticles	preoperative tumor diagnosis	Human epidermal growth factor receptor 2	T2	(66)
Superparamagnetic iron oxide nanoparticles	Glioblastoma	Epidermal growth factor receptor deletion mutant	T2	(87)
Superparamagnetic iron oxide nanoparticles	Differentiate infantile hemangioma	Glucose transporter protein 1(GLUT1) antibody	T2	(88)
Superparamagnetic iron oxide nanoparticles	Prostate cancer	Extracellular epitope of PSMA	T2	(89)
Superparamagnetic iron oxide nanoparticles	Breast Cancer	HER2	T2	(90,91)
Superparamagnetic iron oxide nanoparticles	Hepatocellular carcinoma	AFP and GPC3 antigens	T2	(92)
Superparamagnetic iron oxide nanoparticle coated with amphiphilic polymers and PEGylate	Cancer	Glycoprotein-72 (TAG-72)	T2	(93)
Magnetic-fluorescent iron oxide-carbon hybrid nanomaterials	Breast cancer	CD44	T2	(94)
Dextran-coated superparamagnetic iron oxide	Cancer	PAP2a	T2	(95)
Amphiphilic polymer-coated magnetic iron oxide nanoparticle	Heterogeneous ovarian cancer	HER2	T2	(96)
2,3-Dimercaptosuccinicacid modified superparamagnetic iron oxide nanoparticles	Malignant lymphoma cells	CD20	T2	(97)
Hydroxyethyl starch-coated iron oxide nanoparticles	Cancer	GD2 antigen on neuroblastoma	T2	(98)

Manganese oxide nanoparticles, which shortened T1 relaxation times, are the most commonly used manganese-based nanoparticles in MRI imaging in preclinical studies. Manganese oxide nanoparticles can be synthesized and conjugated with anti-CD105 antibody TRC105, which can target CD105, an antigen exclusively expressed on proliferating endothelial cells (99). In addition to manganese oxide nanoparticles as MRI contrast agents, mesoporous silica nanoparticles can also be used as the framework with manganese oxide nanoparticles because they enable manganese oxide nanoparticles to be easily accessible to water molecules, improving their contrast enhancement in MRI (100). Manganese oxide–mesoporous silica nanoparticles can be conjugated with prostate-specific membrane antigen (PSA) antibodies for targeted prostate cancer detection (Table I). T1 values of manganese oxide mesoporous silica nanoparticles functionalized with PSA antibody are much more significant than manganese oxide mesoporous silica nanoparticles themselves, especially 24 h after administration (82).

Superparamagnetic iron oxide nanoparticles (IONPs) are also widely used as nanoparticle-based MRI contrast agents; and it is commercially available for clinical use for gastrointestinal imaging (Gastromark™, generic ferumoxsil) (101). Based on IONPs’ optimal characteristics for MRI imaging, its applications have been expanded preclinically for systemic administration and tumor imaging. A wide variety of surface chemistries have been reported for this class of nanoparticles, including the attachment of polyethylene glycol (PEG, the process commonly known as “PEGylation”) (93,102), amphiphilic polymers (93,96), and 2,3-dimercaptosuccinicacid (97). Surface modification, such as PEGylation, can help to facilitate effective surface functionalization for antibody conjugation (102).

**Fig. 5:**
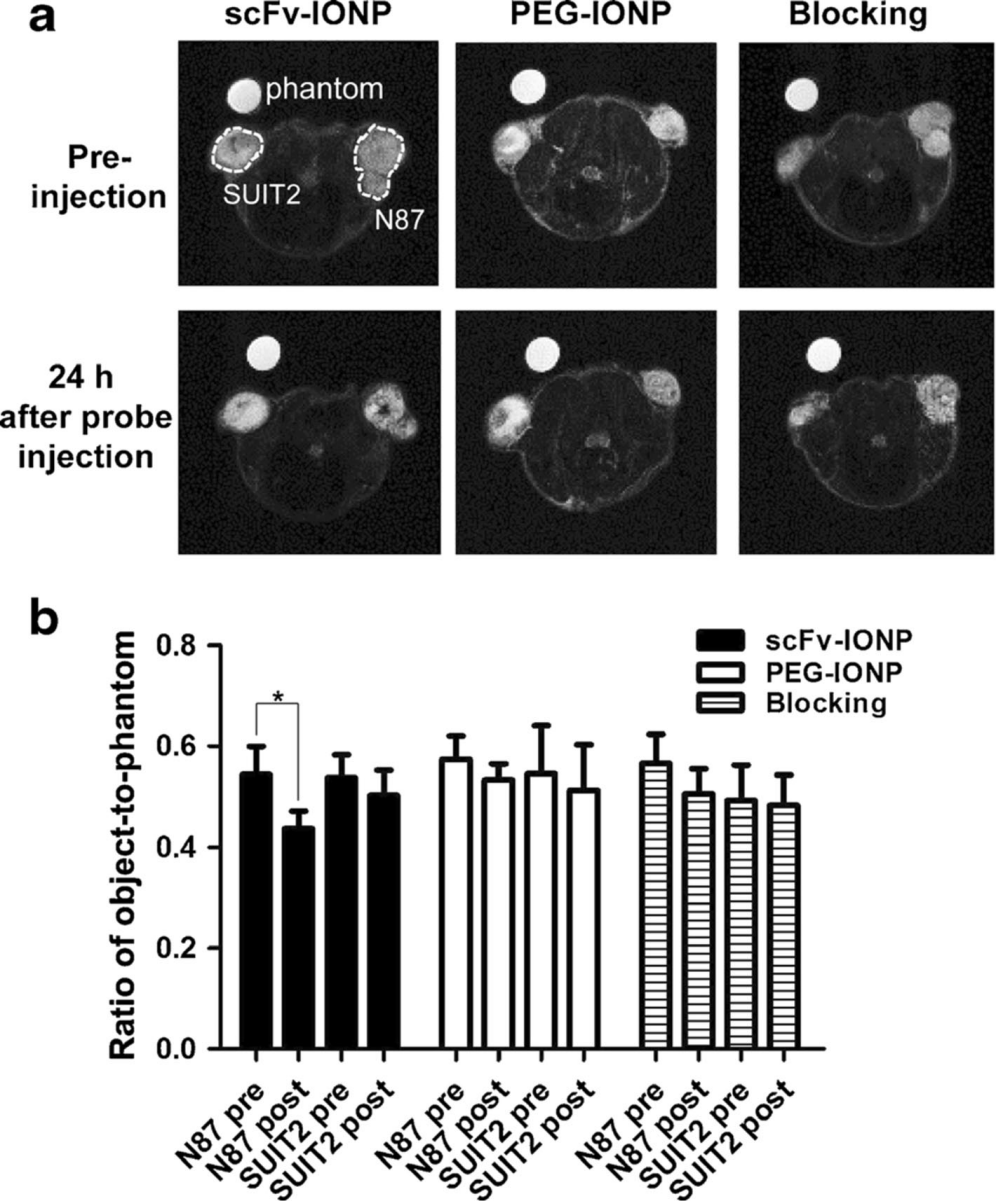
*In vivo* MR tumor imaging post i.v. administration of scFv-IONPs or PEG-IONPs in N87 or SUIT2 tumor-bearing mice. **a** *In vivo* MR images (axial) of scFv-IONP, PEG-IONP, or scFv-IONP mixed with trastuzumab in N87 (HER2+) and SUIT2 (HER2−) bearing mice. **b** Signal intensity of tumors shown as the ratio of object-to-phantom. *p < 0.05 (66)

From a preclinical perspective, IONPs have been widely studied as possible systemic contrast agents for parenteral administration. When combined with the advantages of high sensitivity from IONPs and selectivity from antibodies, antibody-conjugated IONPs became promising contrast agents for early-stage cancer detection (103). Human epidermal growth factor receptor 2 (HER2) amplification or over-expression has been shown to play an important role in the development and progression of certain types of breast cancer. In recent years, HER2 has become an important biomarker and target of therapy for approximately 30% of breast cancer patients (104). HER2 antibodies conjugated to IONPs can selectively bind to HER2-expressing cells, increasing conjugate retention at the site of interest (105). N87 tumor-bearing mice intravenously injected with scFv-IONPs showed a statistically significant difference between pre- and post-injection compared to the PEG-IONP control group, as shown in Fig. 5 (66). MR signal throughout the tumor tissues appeared to be heterogeneous, as shown in Fig. 5a, which indicated the heterogeneous intratumoral distribution of the nanoparticles. Issues with the heterogeneous intratumoral distribution of nanoparticles are a known complicating factor in nanomedicine, as mass transport through the tumor tissue can be hindered by tumor-related factors such as extracellular matrix composition and tumoral interstitial fluid pressure (106,107) besides nanoparticle characteristics.

### Observing Enhanced Cellular Internalization, Tumor Retention, and Accumulation of Antibody Nanoparticle Conjugates

Antibody-nanoparticle conjugates can be employed to enhance nanoparticle-cellular interactions and potentially increase tumor retention for enhanced imaging capabilities. Considering tumor accumulation is a kinetic phenomenon resulting from a balance between nanoparticle extravasation from blood vasculature into tumor tissue and lymphatic drainage, increasing cellular interactions and internalization of nanoparticles can assist in reducing drainage post extravasation. Nanoparticle-antibody conjugates are more likely to be internalized by tumor cells, increasing their residence time in the tissue. As a consequence of increased residence time in the tumor site, visualization of tumors is facilitated when employing multimodal imaging methods, even as nanoparticles are removed from the blood circulation. This is a feature that cannot be achieved with small-molecule contrast agents. This allows for easier logistics between contrast administration and imaging, improvement of imaging signal, and reduction of the need for multiple contrast agent dosing.

Gold nanoparticles conjugated with antibodies have been used to enhance imaging quality and provide anatomical information on tumor tissues preclinically. *In vivo* functionality (83–85) and tissue specificity (86) make gold nanoparticles and gold nanoparticle conjugates exceed the performance of conventional CT contrast agents due to both enhanced signal attenuation and tissue retention (87). The most remarkable sites of accumulation—besides tumor tissues—were in reticuloendothelial system organs, such as liver and spleen, for both naked gold nanoparticles and conjugates. This is expected behavior for foreign particulate systems in the blood circulation and it has been widely described for parenterally administered nanoparticles (88,89). Antibody conjugation with gold nanoparticles leads to optimal cellular uptake, which is an essential factor leading to the enhancement in tumor retention observed preclinically. Radiolabeled PEGylated gold nanoparticles conjugated with cetuximab, an antibody-targeting epidermal growth factor receptor, showed rapid and high cellular uptake in A549 cells, a cell line displaying high EGFR expression, with an average of 14.8-fold increase comparing to naked gold nanoparticles. Moreover, PEG surface modification was used to provide the enhanced blood circulation time of nanoparticles by reducing adsorption of opsonin proteins in the circulation, thus reducing non-specific uptake by macrophages (90). Furthermore, microdistribution studies revealed increased antibody-mediated endocytosis of cetuximab-nanoparticle conjugates in A549 xenografts, whereas naked nanoparticles were retained mainly within the interstitium of the tumor tissue and therefore readily available for lymphatic drainage. These studies highlight how nanoparticle-antibody conjugates can facilitate nanoparticle-cell interactions and foster nanoparticle internalization by the host cell, leading to higher imaging contrast by increasing the retention of the nanoparticles within the tumor tissue (90). This study demonstrates the potential of increasing nanoparticle retention in tumor tissues by fostering their interaction with cancer cells and subsequent internalization. In this case, the retention of nanoparticles within the tumor tissue was verified beyond the observed circulation time of the formulation (urine, liver, and spleen signals decrease while tumor signal remains relatively stable).

**Fig. 6:**
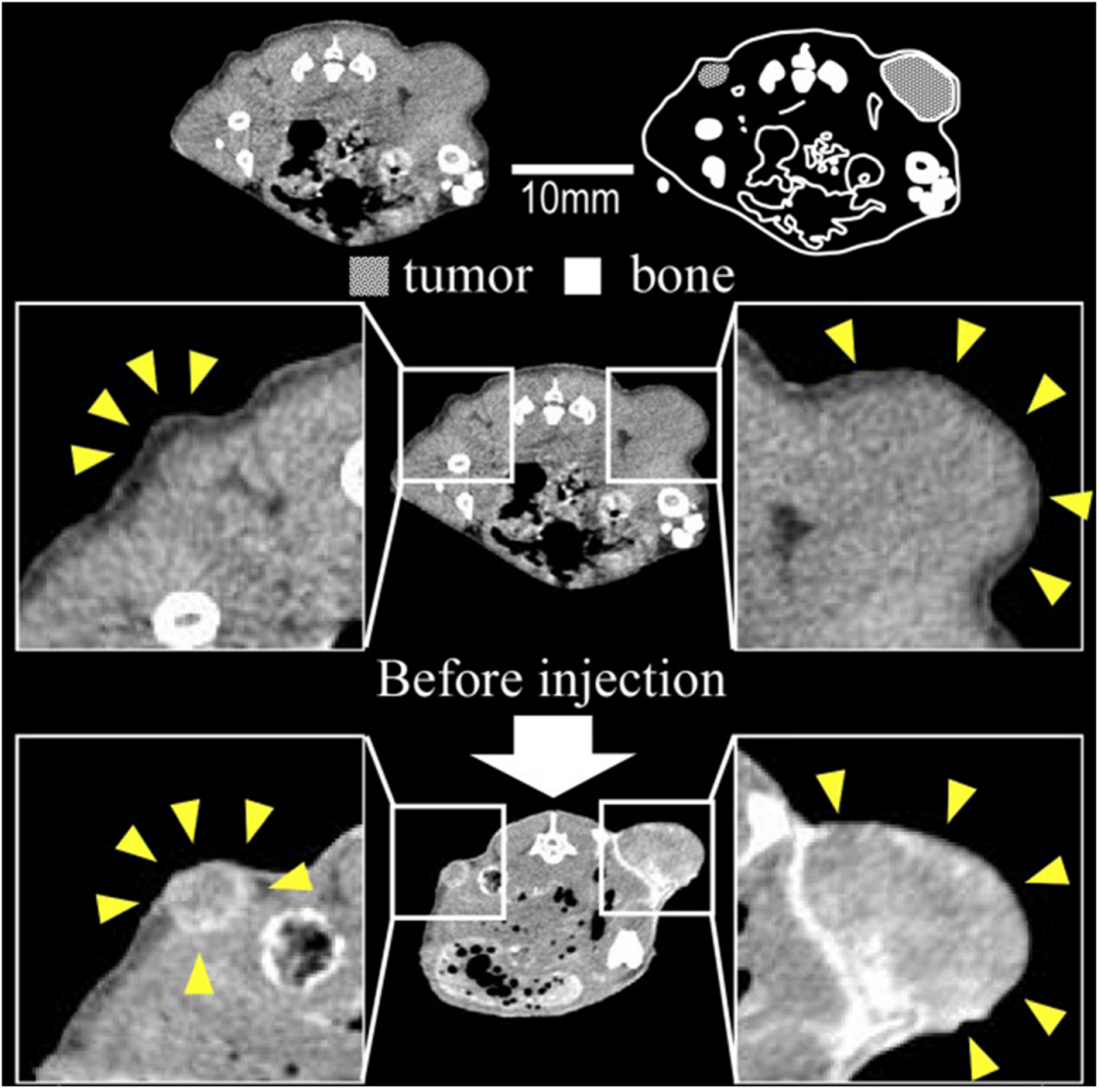
X-ray computed tomography images of KPL-4 murine xenografts demonstrating tumor contrast post 15 nm anti-HER2 gold nanoparticles intravenous administration (108)

Gold nanoparticles coated with PEG and conjugated with anti-HER2 trastuzumab antibodies can be prepared with finely-tuned particle sizes for improved intratumoral distribution (15–30 nm) and led to a two-fold increase in KPL-4 xenograft tumor contrast when compared with images obtained with an iodinated small-molecule contrast agent in X-ray CT, although the majority of the accumulated dose was retained within neovascular regions of the tumor tissues (Fig. 6) (91). In general, smaller particle sizes (15 nm) with HER-2 conjugation led to a 2-fold increased tumor accumulation and retention when compared with larger (30 nm) naked nanoparticles. The small particle size of these gold nanoparticles has been linked to increased extravasation and enhanced retention, but the actual contribution of the targeting ligand under this circumstance is unclear. Although the overall trend shows that antibody-conjugation increases the total dose retained in tumor tissues, when nanoparticles of similar size were compared, no statistical differences between total gold nanoparticle accumulation between targeted and non-targeted particles were found. Similar conclusions have been found in other studies using anti-HER2 gold nanoparticles, where an overall 1.6-fold increase in nanoparticle accumulation in overexpressing HER2 xenografts could be noticed when compared with HER2 negative xenografts (31), but still not too significant when compared with nonspecific distribution in muscles (22-fold higher), which indicates that the overall accumulation may be more likely associated with the nanoparticle size with minor contributions from the targeting ligand.

In general, increased gold conjugate retention in tumor tissues has been attributed to both the longer circulation time of gold nanoparticles, small particle size which enables improved tumor penetration and distribution, and increased retention in the tumor tissue due to antibody interactions with targeted cells. However, the contribution of the former appears to be less relevant when compared with nanoparticle size for the majority of preclinical studies. Conflicting results are found in the literature, indicating the inconsistency of product success when nanoparticle retention is solely attributed to the use of targeting antibodies. Considering the complexity of systemic nanoparticle delivery to tumors, it appears a variety of factors can significantly influence the efficacy of antibody targeting for retention purposes, mainly nanoparticle size, antibody density on the surface of the particle, antibody configuration, as well as antigen expression levels on the targeted cell; and these parameters must be considered concurrently while designing nanoparticle-antibody conjugates.

### Tracking the Tumor Accumulation of Targeted Ultrasmall Nanoparticles and Identifying the Intratumoral Distribution

Nanoparticle accumulation, distribution, and retention in tumor tissues are generally dependent on particle size. Although the majority of nanoparticle systems studied for tumor accumulation display enhanced cellular uptake *in vitro*, these systems usually have failed to translate such increase *in vivo* to the same magnitude. Although there is an incomplete understanding of particle extravasation, diffusion, and internalization within tumors, it is generally understood that smaller nanoparticles may exhibit increased tissue diffusion (92). Ultrasmall gold nanoparticles and silica nanoparticles have been developed to enhance accumulation and retention at the target site, also improve tissue penetration and diffusion of targeted nanoparticles to effectively achieve a targeting effect. More importantly, although ultrasmall nanoparticles may enhance tissue extravasation, these particles are also more easily removed from the tissue due to lymphatic drainage and vascular backflow associated with high tumoral interstitial fluid pressures. This limitation can be overcome by employing targeting ligands that enhance the residence time and retention of ultrasmall particles within the tissue.

Nanoparticles within a size range below 10 nm are complicated to visualize using imaging modalities such as X-ray CT and MRI due to sensitivity and resolution limitations but may be easily visualized using radiotracers *via* positron emission tomography (PET). Nanoparticles can be directly tagged with high specific activity radiotracers to provide real-time imaging *in vivo* at much lower concentrations when compared with other imaging modalities. Photons emitted by a radionuclide such as ^64^Cu, ^89^Zr, ^13^N, ^18^F, ^72^As, or ^68^Ga can be detected by a scanning device, typically scintillator crystals coupled to a photomultiplier (94). Furthermore, hyphenated techniques have been developed to promote multimodality imaging within the same equipment (e.g., PET-CT, PET-MRI) and facilitating anatomical identification and co-registration of functional imaging data (PET) with anatomic imaging (CT/MRI) (95).

In this sense, antibody-nanoparticle conjugates can further increase the sensitivity and specificity of PET but may also confer additional functionalities to radiotracers beyond the typical application as a molecular marker. With exquisite specificity, antibodies can be labeled by radionuclides and synthesized to target specific ligands instead of relying solely on the tissue uptake of a radiotracer for biochemical processing. Simply radiolabeling antibodies—although technically feasible—may prove challenging from a pharmacokinetic perspective upon clinical administration as, if unaccompanied by other vehicles, antibodies can be easily removed from the body. Nanoparticles have the potential to act as contrast agents for PET in cancer imaging by delivering encapsulated radiotracers to tumor tissues, and ultra-small nanoparticles are specially relevant for this application due to their improved intratumoral distribution when compared with larger nanoparticles. For molecular imaging applications, low specificity and heterogeneous tumor penetration can lead to low PET resolution (98), and therefore enhanced intratumoral distribution is desired as ultra-small radiotracer-containing nanoparticle-antibody conjugates display potential for targeting and increasing tissue retention of nanoparticles, while providing high specificity to ligands and low off-target accumulation in non-targeted tissues (109).

A single-chain variable fragment (scFv) format of the HER2-targeting antibody Trastuzumab, which is the first FDA-approved monoclonal antibody for the treatment of metastatic breast cancer, can be used to functionalize ultrasmall silica nanoparticles (5–10 nm), namely C’ dots (110). The anti-HER2-targeted immunoconjugate with ultrasmall fluorescent core–shell silica nanoparticles were labeled by ^89^Zr. A multi-step linking strategy that can conjugate radiometal chelators, click chemistry functional groups, and anti-HER2 scFv fragments was conducted, providing a controllable and scalable nanoparticle platform for targeted PET imaging while still sustaining an ultra-small particle size (< 10 nm) desired for improved intratumoral distribution. Furthermore, the small particle size ensures the bulk number of injected nanoparticles is removed from the circulation *via* renal filtration instead of mainly accumulating in the liver and spleen.

**Fig. 7:**
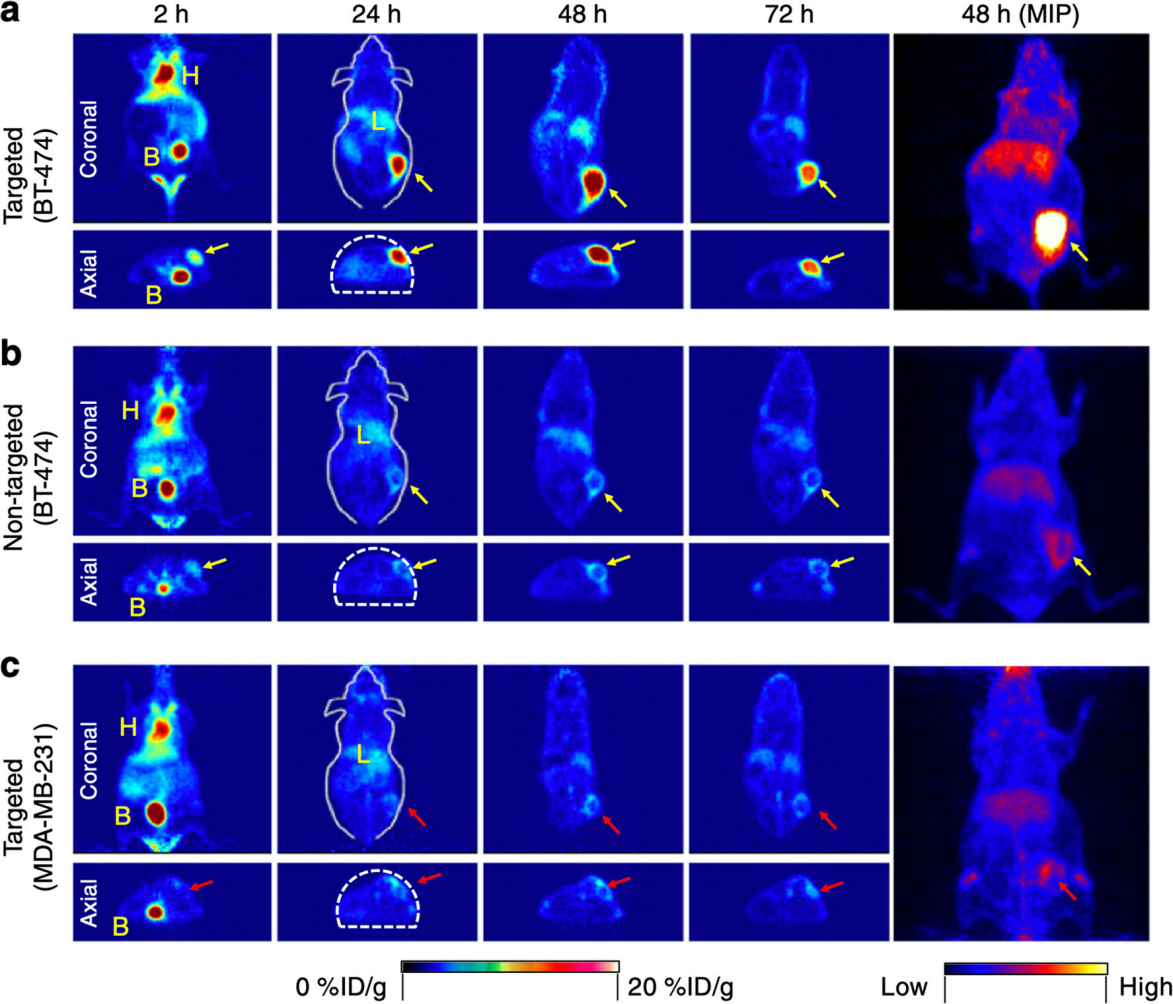
*In vivo* PET imaging in xenograft breast cancer models following administration of HER2-targeted radiolabeled C’ dots (i.v.). Serial coronal and axial tomographic PET images acquired at 2, 24, 48, and 72 h post i.v. injection. **a** Targeted group: ^89^Zr-DFO-scFv-PEG-Cy5-C’ dots in BT-474 tumor model, **b** non-targeted group: ^89^Zr-DFO-Ctr/scFv-PEG-Cy5-C’ dots in BT-474 tumor model, and **c** targeted group: ^89^Zr-DFO- scFv-PEG-Cy5-C’ dots in MDA-MB-231 tumor model. *H* heart, *B* bladder, *L* liver (121)

Typical PET images collected with C’ dots are shown in Fig. 7. Ultrasmall anti-HER2 fluorescent core–shell silica nanoparticles not only showed great enhancement on accumulation and retention at the target site but also improved target tissue penetration and diffusion, likely associated with their ultrasmall particle size (< 10 nm), overcoming the limitation of nanoparticle distribution in perivascular tumor cells commonly observed for larger nanoparticles. The kinetic improvements in tumor distribution are clearly seen in Fig. 7, where the bulk signal from injected nanoparticles is reduced 24 h post-injection, whereas the tumor signal remains significantly high for up to 72 h. These ultrasmall nanoparticles are able to penetrate into and distribute throughout the tumor tissue, co-localizing with HER-2 expression detected by *ex vivo* immunohistochemical staining, providing an optimal platform for molecular imaging and antigen detection in real time. In contrast, for HER-2 low expression tumors, the signal was diminished and predominantly localized along the tumor periphery and seen within stromal tissue (Fig. 7c).

Cetuximab, a monoclonal antibody that binds to the epidermal growth factor receptor (EGFR), has also been studied as a targeting tool to functionalize small gold nanoparticles. Because of the over-expression of EGFR in many epithelial solid tumors, EGFR-functionalized gold nanoparticles labeled by ^89^Zr displayed high tumor contrast in a metastatic colorectal cancer model as a result of selective accumulation and retention of these nanoparticles in the tumor tissue. Considering one of the main challenges with antibody conjugates is to ensure full antibody functionality, the authors demonstrated preserved EGFR recognition ability of cetuximab after chelation and radiolabeling, as demonstrated by a tumor-to-background noise ratio nearly four times higher than blocking group (111).

Overall, the use of ultrasmall nanoparticles enables enhanced intratumoral distribution beyond perivascular regions and may provide an optimal platform to investigate the presence of a wide variety of antigens within the tumor tissue. Considering their small size and easier removal from the tissue, nanoparticle retention as a function of antibody functionalization may be used to estimate the presence and density of antigens within the tissue without need for invasive procedures (i.e., biopsy) and immunohistochemistry.

## CURRENT CHALLENGES AND FUTURE PERSPECTIVES

Nanoparticle-antibody conjugates possess significant advantages for *in vitro* diagnostics regarding the increase in sensitivity and reliability of immuno-based assays (35,112). Improvements in analytical performance are evident due to highly tunable nanoparticle chemistries associated with surface plasmon resonance effects. Considering the wide applications of immunoassays and their potential of enabling point-of-care testing with relatively low cost, improvements on these methodologies are highly desired especially in resource-poor areas. Nonetheless, the translation of nanotechnology-enabled immunoassays from laboratory to clinical settings still faces significant challenges from manufacturing and commerciality perspectives. There are still questions regarding the real commercial value of this approach as immunoassays are inherently sensitive and specific, and nanoparticle-antibody conjugates mainly play a role in amplifying the signal and further increasing detection sensitivity. For most biological analytes of clinical interest, adequate sensitivity is already achieved with current commercial immunoassay technologies. Under such circumstances, diagnostic industries must balance the additional cost of implementing a new technology *versus* the current clinical need.

From a manufacturing perspective, nanomaterials are known for manufacturing reproducibility and scaling-up issues, besides generally displaying physical stability limitations (113). These factors can hinder the manufacturing of consistent products with adequate physicochemical characteristics for the desired product performance. Furthermore, antibody-nanoparticle conjugates may also be subjected to conjugation instabilities, further increasing manufacturing complexity and final product cost. In this sense, novel technologies are enabling large-scale continuous manufacturing of nanomaterials with greater control, precision, and reproducibility, potentially addressing most of the manufacturing challenges associated with nanoparticle-based formulations (114–116). Custom-built continuous manufacturing setups have been effective in improving the state of the art of nanoparticle manufacturing, but the incorporation of an antibody conjugation step into these systems is yet to be achieved.

On the other hand, *in vivo* applications of nanoparticle-antibody conjugates for imaging purposes are still at a preclinical level. *In vivo* applications in the clinic have been hindered by not only manufacturing issues discussed above but also limited product performance due to poor clinical translation. In general, there are toxicity concerns when nanoparticles are administered systemically (108) and scientists are yet to fully understand the long-term biological impact of a wide variety of nanomaterials. Furthermore, due to their relatively large size, nanoparticles show biodistribution patterns that are significantly different when compared with a small-molecule imaging agent or a free antibody, reducing or completely hindering nanoparticle interactions with the tissue of interest.

In principle, nanoparticle-antibody conjugates are expected to show increased targeting ability and higher accumulation in tissues of interest due to the functionality of antibodies. This is an expected outcome based on vast antibody knowledge, *in vitro* studies, and well-controlled *in vivo* preclinical studies. In reality, a wide variety of circumstantial factors influence this expected product performance leading to significant translational issues. For instance, access to tissues from the blood vasculature, interaction competition, and effective clearance of nanoparticles by the reticuloendothelial system has proven to offer exponential obstacles for adequate product performance *in vivo* (117). These are issues that have surrounded nanoparticle-based drug delivery to tumors for decades and are also present in nanoparticle-based imaging. Non-specific reticuloendothelial system uptake can be reduced by employing ultrasmall nanoparticles, which favor renal filtration, but this approach may not be feasible for every nanoparticle technology and payload. Furthermore, nanoparticle distribution within the tumor tissue beyond perivascular regions is challenging for most nanoparticles due to mobility limitations within the extracellular matrix associated with particle size.

Tumor imaging is a major focus application for antibody-nanoparticle conjugates. Nanoparticles for this application typically take advantage of either active (e.g., antibody conjugation on the surface of the nanoparticles) and/or passive targeting (i.e., enhanced permeation and retention effect). However, only a small fraction of the injected nanoparticle-based dose is expected to accumulate in solid tumor tissues, whereas the vast majority of the dose exhibits non-specific accumulation and elimination (1,2). This poor tumor accumulation has been associated with a wide variety of tumor-related issues (intra/transcellular transport, intrinsic variabilities associated with enhanced permeation and retention effect, as well as the influence of nanoparticle physicochemical characteristics on their transport within the tumor stroma) and biological clearance (2). Due to their macromolecular nature, nanoparticle extravasation into tumor tissues is directly related to the degree of tumor growth and maturation. In general, tumor vasculature becomes increasingly permeable to macromolecular structures as the tumor grows exponentially. Leaky vessels are generally concentrated at the tumor-host interface and within the stroma between tumor nodules (118), whereas tumor-penetrating vessels usually exhibit little leakage of macromolecular structures (118). Therefore, nanoparticle extravasation, distribution, and retention into solid tumor tissue are directly related to the inherent characteristics of the tumor vasculature at a given time. That means nanoparticle-based contrast agents may fail to promote visualization of small tumor tissues which do not display optimal vascular characteristics for the EPR effect, regardless of the presence of a targeting antibody. These issues confer significant consequences on the translation of nanoparticle-based imaging agents for human use and must be fully assessed and considered to increase the likelihood of product success. Furthermore, most targeting antibodies employed in conjugate preparations do not target vascular ligands, and therefore these antibodies will mainly assist in increasing tumor retention but not necessarily tumor extravasation, which still relies on passive accumulation.

Considering the difficulties associated with tailoring the pharmacokinetics of nanoparticles, the best path forward is to take advantage of these limitations to invent a new generation of imaging agents with specific functionalities, leveraging the natural *in vivo* kinetics of particulates in blood circulation instead of seeing it as a limitation for applications for which this approach is not ideal. For instance, nanoparticle-based imaging agents could be employed to investigate the likelihood of nanoparticle accumulation in certain tissues in the clinic, offering imaging data that could enable imaging-guided therapies (e.g., assessing tumor leakiness in patients for treatment selection, or commonly referred as “EPR imaging”). This approach does not focus on imaging the tumor tissue as a whole, but rather focuses on imaging the extravasation of nanoparticles into the tissue regardless of the extent of accumulation, and the information obtained will be valuable for treatment strategy determination.
